# miR-212 Promotes Cardiomyocyte Hypertrophy through Regulating Transcription Factor 7 Like 2

**DOI:** 10.1155/2022/5187218

**Published:** 2022-08-24

**Authors:** Jinxia Yuan, Guoliang Yuan

**Affiliations:** Department of Cardiology, Shuyang Hospital of Traditional Chinese Medicine, Yangzhou University, Shuyang, Jiangsu, China

## Abstract

To explore the role and possible mechanism of miRNA-212 in heart failure (HF). The rat model of abdominal aortic constriction was constructed, the changes of myocardial morphology were observed by hematoxylin-eosin (HE) staining, and the hypertrophy-related marker molecules were detected by quantitative real-time polymerase chain reaction (qRT-PCR). At the cellular level, phenylephrine and angiotensin II were added to induce cardiomyocyte hypertrophy. The overexpression of miR-212 adenovirus was constructed, and the expression of miR-212 was overexpressed, and its effect on cardiac hypertrophy (CH) was detected by immunofluorescence and qRT-PCR. Then, the mechanism of miR-212 regulating CH was verified by website prediction, luciferase reporter gene assay, qRT-PCR, and western blotting assay. In the successfully constructed rat model of abdominal aortic constriction and cardiomyocyte hypertrophy, ANP and myh7 were dramatically increased, myh6 expression was decreased, and miRNA-212 expression was increased. Overexpression of miRNA-212 in cardiomyocytes can promote cardiomyocyte hypertrophy, while knocking down miR-212 in cardiomyocytes can partially reverse cell hypertrophy. In addition, miR-212 targets TCF7L2 and inhibits the expression of this gene. miRNA-212 targets TCF7L2 and inhibits the expression of this gene, possibly through this pathway to promote cardiomyocyte hypertrophy.

## 1. Introduction

Cardiovascular disease, especially heart failure (HF), has always been a serious threat to human health [1]. HF is a syndrome of cardiac structure and function disorder caused by a variety of physiological factors, which is manifested as ventricular systolic ejection or diastolic filling dysfunction, which can lead to clinical symptoms such as sitting breathing, nocturnal paroxysmal dyspnea, lower limb pitting edema, and fatigue [2]. Despite advances in the treatment of HF, its high morbidity and mortality remain important issues affecting public health and socioeconomic development [3]. According to the analysis, HF is one of the main causes of hospitalization in the elderly over 65 years old, and it is one of the primary medical problems to be solved [4]. Of various cardiovascular diseases, it will eventually lead to HF, including myocardial infarction, after pressure overload (aortic stenosis and poor control of high blood pressure), inflammation (myocarditis) and overload capacity of hypertrophic cardiomyopathy (valvular regurgitation), genes inherited cardiomyopathy, and drug factors (such as alcohol and drugs such as cocaine, with heart damage side-effects adriamycin) [5]. The mechanism of HF is a complex signal cascade reaction between cell and molecular physiology change regulation of disease: myocardial cells, myocardial hypertrophy (MH), electrophysiological changes, contraction dysfunction, oxidative stress, metabolic disorders, extracellular matrix remodeling, apoptosis, and necrosis. Finally, it leads to the rational reconstruction of cardiomyopathy, with reduced cardiac output, blood stasis, increased capacity load, increased chronic filling pressure, hypoxia, and edema. With the deepening of the research on the pathogenesis of HF, the regulation of cellular and molecular signals has attracted more and more attention [6].

Noncoding RNA (ncRNA) in the RNA family is an important regulatory RNA, including a variety of different subgroups, such as small sequence microRNA (miRNA, tRNA, snRNA, and snoRNA), large sequence long noncoding RNA (lncRNA), and circularRNA (circRNA), which can participate in the HF process as important regulatory factors [7]. miRNA genes are highly conserved in evolution and can be transcribed as independent transcription units in the intergenic or intron and exon regions of protein-coding genes [8]. Studies have shown that miRNAs play a crucial regulatory role in every link of the occurrence and development of heart diseases, participating in and regulating angiogenesis, MH, myocardial cell and interstitial fibrosis, inflammatory response, apoptosis, and necrosis [9].

The pathologic process of HF involves CH. MH in cell volume increases; protein secretion increased as the characteristics of the pathological process of sarcomere organization; its potential triggers for hemodynamics, ischemic injury and nerve endocrine imbalance, a variety of pathological conditions, such as its internal mechanism for fetal gene expression, excitation contraction coupling changes, energy metabolism, balance, molecular mechanisms involved in Ca^2+^ depend on the way, and PI3K/Akt pathway [10]. Studies have shown that many miRNAs function in a positive or negative way to regulate the molecular mechanisms of CH. For example, the expressions of miR-23a, miR-27b, miR-125b, and miR-195 were upregulated in the early stage of CH caused by aortic stenosis [11], while the expressions of miR-23a, miR-23b, miR-24, miR-125b, miR-195, miR-199a, and miR-214 were upregulated in the later stage, while the expressions of miR-93, miR-133a, miR-150, and miR-181b were downregulated [12], while miR-34a, miR-28, miR-148a, and miR-93 were upregulated and miR-126 and other miRNAs were downregulated in right HF hypertrophy caused by pulmonary artery stenosis [13]. Current studies have shown that miR-1, miR-18, miR-19, miR-133, miR-378, miR-185, and miR-155 can inhibit various types of CH, while miR-208 family, miR-100, miR-125b, miR-214, miR-212, miR-23, miR-24, miR-195, and miR-199a can promote CH [14].

Currently known diagnostic markers of HF are BNP and NT-proBNP, which have high sensitivity but low specificity. With the in-depth study of miRNA, miRNA may become a new marker of HF. Most miRNAs exist in cardiomyocytes but can also be detected in body fluids (serum, plasma, and urine), which are specifically associated with the physiological process of HF and have high specificity [14]. Fukushima et al. found that plasma expression of miR-126 in patients with chronic HF with low expression of NT-proBNP was elevated, suggesting that it might be used as a marker of HF [15]. Other studies found that the expression levels of miR-18a-5p, miR-26b-5p, and miR-30b were decreased in acute HF, while miR-499 was highly expressed [14]. However, due to limited factors such as small sample size and lack of standardization of detection methods, the diagnostic role of miRNA still needs to be further explored. miRNA plays an important regulatory role in posttranscriptional level and is therefore a potential therapeutic target.

Studies have shown that miR-212 plays a role in promoting CH, but its mechanism has not been fully revealed [16, 17]. This research selects miR-212 as the research object to explore the function and mechanism of miRNA-212 in the process of MH.

## 2. Materials and Methods

### 2.1. Animal Experiment

The SD rats, weighing about 200-220 g, were purchased from Weitonglihua Company (Beijing, China) and fed in the animal center of our hospital. Rats were fasted and watered for 12 hours after adapting to the environment after 3 days of regular feeding. The abdominal cavity was opened in a sterile environment, and the abdominal aorta was obtuse separated from the fascia with forceps. The separated abdominal aorta was bound and tied with 0.6 mm needle tube (Jiancheng, Nanjing, China) with silk thread, the needle tube was removed, and the abdominal posterior suture skin was closed. The abdominal aorta was not ligated in the sham group, and the rest of the operations were the same. After feeding the rats for 4 weeks, the cardiac function of the rats was examined by echocardiography (Philips, Eindhoven, The Netherlands) before the rats were sacrificed. After anesthesia, the rat heart was quickly removed and isolated from the left ventricle. The blood was thoroughly washed with phosphate-buffered saline (PBS) solution (Beyotime, Shanghai, China) to remove the blood and stored in the -80°C refrigerator for later use. This study was approved by the Animal Ethics Committee of Shuyang Hospital of Traditional Chinese Medicine Animal Center.

### 2.2. Cell Culture

Newborn suckled rats were sterilized with 75% alcohol (Jiancheng, Nanjing, China) on the skin surface of the suckled rats for 1-3 days. The skin under the costal margin of the suckled rats was cut off in the super clean table, the heart was exposed, and the forceps were removed. It was quickly placed in the precooled D-Hanks solution (Elabscience, Wuhan, China), the atria were removed, the blood in the heart was washed, and the ventricles were cut into small pieces with the size of <1 mm^3^ by ophthalmic scissors (Belevo (Beijing) Medical Technology Co., Ltd., Beijing, China), and the blood was further washed. The obtained myocardial tissue was placed in a petri dish with a diameter of 6 cm, diluted with 1% collagenase (Elabscience, Wuhan, China) and 1x D-Hanks solution (Elabscience, Wuhan, China) in a ratio of 1 : 4, and placed in a refrigerator at 4°C. After 15 hours, the tissue block was blown into turbid suspension with a pipette. The obtained suspension was added to a 15 mL centrifuge tube, followed by a complete Dulbecco's Modified Eagle's Medium (DMEM; Life Technology, Wuhan, China) culture solution to terminate digestion. The suspension was centrifuged at a speed of 1000 rpm for 5 min, and the supernatant was removed. The cells were added with a complete DMEM culture solution of 10 mL and transferred to a 10 cm culture dish. After 1 hour, the culture medium was removed and gently knocked and shaken. The culture medium was transferred to another 10 cm culture dish. The previous process was repeated. After 24 hours, Brdu (Elabscience, Wuhan, China) was added at a concentration of 1% to inhibit the proliferation of nonmyocardial cells, and the culture was continued in a CO_2_ thermostatic incubator. 48 hours after the replacement of DMEM solution (excluding serum and streptomycin), further ensure consistency of myocardial cells. Under the condition of myocardial cells in serum-free culture after 12 hours, the concentration of 100 microns to join phenylephrine (PE, Tianpu Biochemical Pharmaceutical, Guangzhou, China) and final concentration tendency for 1/L Ang II (Tianpu Biochemical Pharmaceutical, Guangzhou, China) stimulate myocyte; after 48 hours in fluorescence microscope cell morphology, extract RNA and save for experiment in -20°C refrigerator.

### 2.3. Western Blotting Technology

The cultured plate cells were washed with PBS, 50-70 *μ*L radioimmunoprecipitation assay (RIPA; Thermo Fisher Scientific, Waltham, MA, USA) lysate was added to each well, and the ice abrasive rod was ground for 20 s for 3 times, followed by 30 s of vortex homogenate. The cells were left on the ice for 5 min and then centrifuged for 3 times at 4°C for 30 min at 14000 × g. The supernatant was taken, and the total protein concentration was determined by the bicinchoninic acid (BCA) protein concentration measurement kit (Jiancheng, Nanjing, China). Total protein loading of 20~50 *μ*g was separated by sodium dodecyl sulphate-polyacrylamide gel electrophoresis (SDS-PAGE), and the separated protein was transferred to a polyvinylidene difluoride (PVDF; Thermo Fisher Scientific, Waltham, MA, USA) membrane. After 5% of the skim milk powder was sealed for 1 hour, it was incubated overnight with the corresponding primary antibody (TCF7L2, Abcam, Cambridge, MA, USA, Rabbit, 1 : 2000; GAPDH, Proteintech, Rosemont, IL, USA, 1 : 2000) at 4°C, washed with 0.1% PBST solution, and coincubated with the secondary antibody (goat anti-rabbit IgG antibody, Yifei Xue Biotechnology, Nanjing, China, 1 : 2000), and the enhanced chemiluminescence (ECL) technology was used to develop western blot bands, and the grayscale analysis software was used for quantitative analysis.

### 2.4. Quantitative Real-Time Polymerase Chain Reaction (qRT-PCR)

The specific reverse transcription primers of miRNAs were combined with corresponding miRNAs, which were reverse-transcribed into cDNA in the presence of reverse transcriptase reaction system, and the template fragments were amplified. The reaction process was: 16°C, 30 min, 42°C 90 min. After the reverse transcription product was diluted by 40 times, PCR was performed according to the instructions (Thermo Fisher Scientific, Waltham, MA, USA). The reaction process was as follows: 95°C, 20 s; 58°C, 15 s; and 72°C, 20 s, 40 cycles. The reaction system used GAPDH as an internal reference, and the results were analyzed by qRT-PCR. The results were expressed in relative multiples to compare the relative contents of the target molecules in each group. Primers used are shown in Table 1.

### 2.5. Hematoxylin-Eosin (HE) Staining

The removed heart was washed three times with PBS solution and fixed in 4% paraformaldehyde (Sinopharm Chemical Reagent, Shanghai, China) to maintain the original shape of the cells. The tissues were washed with PBS and dehydrated successively with low concentration of alcohol to high concentration of alcohol to gradually remove the water in the myocardial tissue. The alcohol in the myocardial tissue was removed with xylene (Sinopharm Chemical Reagent, Shanghai, China). The tissues were immersed in the melted paraffin wax (Sinopharm Chemical Reagent, Shanghai, China) for about 2–3 hours and then solidified into blocks. Then, we used a slicer to cut the package into thin slices. The sections were soaked in xylene (Sinopharm Chemical Reagent, Shanghai, China) for about 5 min, and then, the sections were soaked in gradient ethanol for many times, each time for about 5 min. After dyeing with hematoxylin (Sinopharm Chemical Reagent, Shanghai, China) for about 10 min, rinsed with hematoxylin, then added acid alcohol for about 10 s for color separation, rinsed with PBS, then dyed with 1% eosin (Sinopharm Chemical Reagent, Shanghai, China) for 10 min, and then washed with distilled water to remove eosin. The pure alcohol was dehydrated again, and the section was made transparent by xylene. Add gum to the transparent slice, cover with the cover glass, and seal the slice with the corresponding label.

### 2.6. Immunofluorescence

After routine isolation, rat cardiomyocytes were inoculated with a density of 1 × 10^5^/ml in a 24-well cell culture dish, in which those stimulated by PE and Ang II were the experimental group and those not stimulated were the control group. DMEM was absorbed and washed with PBS solution for 3 times. Then, 4% paraformaldehyde was added to fix the cardiomyocytes, which were fixed at room temperature for about 30 min. Then, PBS was added to soak them for cleaning, and they were placed in a shaking table for about 15 min. 10% BSA (bovine serum albumin, Thermo Fisher Scientific, Waltham, MA, USA) was sealed at room temperature for 10 min, washed once with PBS solution, and incubated overnight with antibody a-actinin (Abcam, Cambridge, MA, USA, Rabbit, 1 : 1000) at room temperature. Wash with PBS containing 0.1% twain for 3 times, each time for about 10 min. Incubate with fluorescently labeled secondary antibody against light for 2 min. The above steps for cleaning were repeated.

### 2.7. Double-Luciferase Reporter Gene Assay

Complete DMEM was used to culture 293T cells (ATCC, Manassas, VA, USA), Lipofectamine^2000^ (1 UI/hole; R&D Systems, Minneapolis, MN, USA) and opti medium (50 UI/hole) thoroughly incorporated, miR-212 overexpressed plasmid (400 ng/hole; R&D Systems, Minneapolis, MN, USA), opti medium (50 UI/hole) thoroughly incorporated, pGL3 recombinant plasmid (200 ng/hole; R&D Systems, Minneapolis, MN, USA) and opti medium (50 UI/hole) thoroughly incorporated, and let stood for 5 min. The three mixing systems were then thoroughly mixed and left to rest at room temperature for 20 min. Then, they were uniformly added to the cell culture dishes and continued to be routinely cultured in the cell culture box for 24 hours. 293T cells were fully lysed by cell lysate. 10 *μ*L cell lysate was taken, and 40 *μ*L luciferase detection reagent (R&D Systems, Minneapolis, MN, USA) was added to generate firefly fluorescence signal. For fluorescence detector measurement value of firefly luciferase report gene, we finally added 40 *μ*L sea kidney in sample luciferase substrates, blended after 10 s, terminated the above reaction, and started the sea renal luciferase reaction. Finally, we measured the fluorescence signal.

### 2.8. Statistical Analysis

The data were expressed as mean ± standard error. All the experimental results were repeated 3 times. *t*-test was used for comparison between the two groups. We default *P* < 0.05, which is a significant difference.

## 3. Results

### 3.1. Identification of Rat Model of Abdominal Aortic Coarctation and Hypertrophy

**Figure 1:**
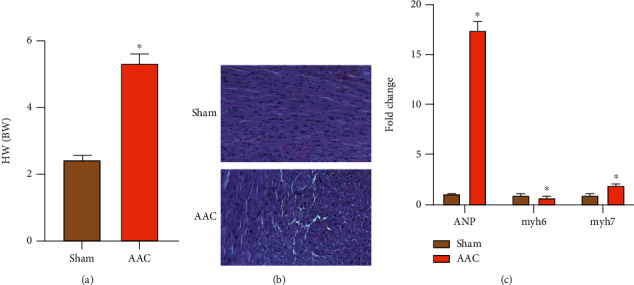
Identification of rat model of abdominal aortic coarctation and hypertrophy. (a) The rate of HW/BW in sham and AAC groups (“∗” indicates statistical difference from the sham group *P* < 0.05). (b) HE staining of renal tissues in sham and AAC groups (200x). (c) The expression of ANP, myh6, and myh7 in the sham and AAC groups (“∗” indicates statistical difference from the sham group *P* < 0.05).

After abdominal aorta ligation, SD rats had increased cardiac afterload, in order to maintain normal blood supply, at the same time, RAAS system was activated, due to increased cardiac systolic protein synthesis, compensatory hypertrophy occurred, and cardiac weight/body weight (mg/g) increased (Figure 1(a)). HE staining of the left ventricle of the heart showed that the appearance of the heart increased dramatically 4 weeks after abdominal aortic ligation. At the same time, the longitudinal section of HE staining showed that, compared with sham group, the left ventricular wall of the hypertrophy group was dramatically thickened, and the left ventricular cavity was reduced, presenting centripetal hypertrophy (Figure 1(b)). During the process of CH remodeling, some fetal genes are expressed again. The commonly used marker molecules in the literature include atrial natriuretic peptide (ANP), myosin heavy chain 6 (myh6), and myosin heavy chain 7 (myh7). The expression level of these molecules can usually reflect the degree of CH or HF. Therefore, in this experiment, we will detect the expression levels of these marker molecules by qRT-PCR. qRT-PCR results showed that compared with the sham group, ANP expression increased dramatically, myh6 decreased, and myh7 increased after 4 w of ligation, which may be related to the compensatory mechanism of the heart (Figure 1(c)). Overall, the results show that the model is successful.

### 3.2. Identification of Primary MH Model

**Figure 2:**
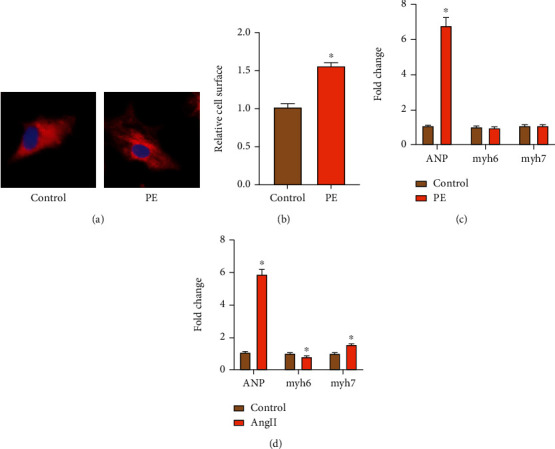
Identification of primary MH model. (a) Cell immunofluorescence staining *α*-actinin in control and PE groups. (b) Analysis of cardiomyocyte surface area (“∗” indicates statistical difference from the control group *P* < 0.05). (c) The expression of ANP, myh6, and myh7 in the control and PE groups (“∗” indicates statistical difference from the control group *P* < 0.05). (d) The expression of ANP, myh6, and myh7 in the control and Ang II groups (“∗” indicates statistical difference from the control group *P* < 0.05).

In this study, we used classical phenylephrine (PE) and Ang II to induce cardiomyocyte hypertrophy. After 2 days of induction by PE and Ang II, the cardiomyocytes beat well. After specificity of a-actinin tags parallel cell immunofluorescence staining, we can see Figure 2(a): stick wall myocardial cell was triangular or fusiform, control cells were relatively flat, PE group of myocardial cells in full and obviously increase the surface area, and in the control group and the PE induced CH group, 100 cells in 10 visual fields were randomly selected and their surface area was measured. In the PE group, the cell surface area was increased compared with the control group (Figure 2(b)). Myocytes were stimulated with 100 *μ*M PE and 10 *μ*M Ang II for 12 h after starvation induction, and RNA was extracted for 48 hours after conventional culture to detect the mRNA expression levels of ANP, myh6, and myh7, respectively. The results showed that the expressions of ANP in PE group and Ang II group were dramatically increased, while the expressions of myh6 and myh7 were not dramatically changed in the PE group (Figure 2(c)), and the expression levels of myh7 were increased in the Ang II group, with statistical differences (Figure 2(d)). Overall, the results indicate successful cell modeling.

### 3.3. miR-212 Promotes CH

**Figure 3:**
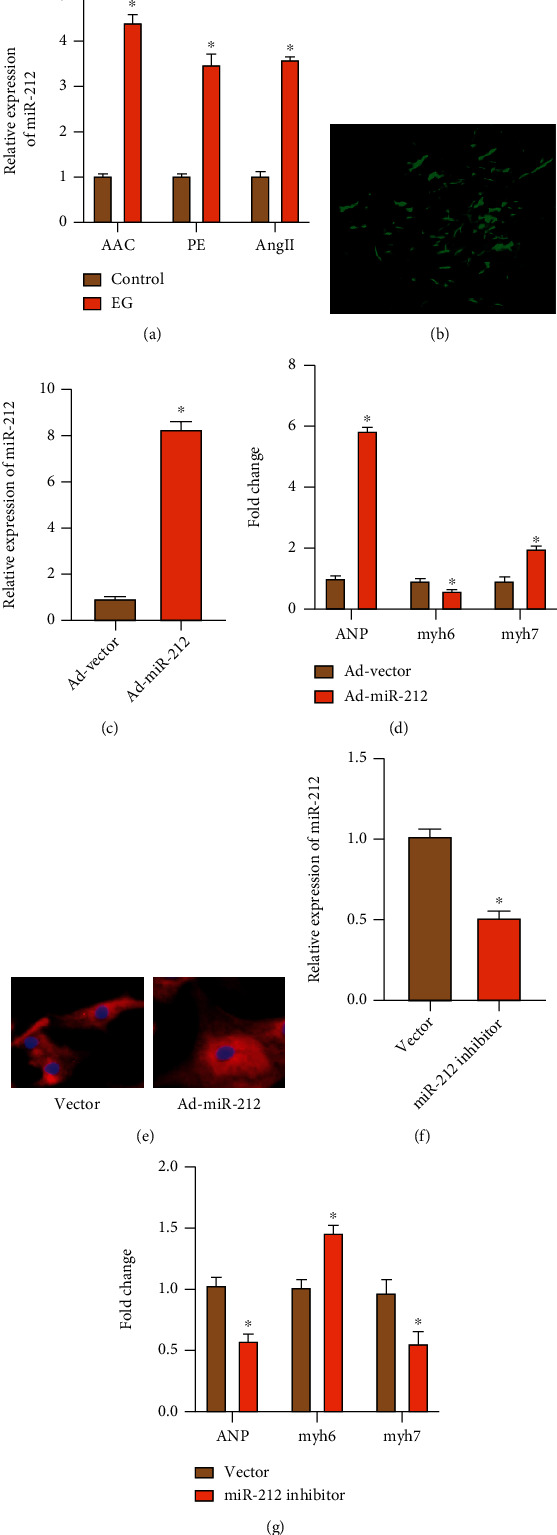
miR-212 promotes CH. (a) The expression of miR-212 in the control and EG groups (“∗” indicates statistical difference from the control group *P* < 0.05). (b) Effect of primary cardiac myocytes transfected with Ad-miR-212 adenovirus. (c) The expression of miR-212 in the Ad-vector and Ad-miR-212 groups (“∗” indicates statistical difference from the Ad-vector group *P* < 0.05). (d) The expression of ANP, myh6, and myh7 in the Ad-vector and Ad-miR-212 groups (“∗” indicates statistical difference from the Ad-vector group *P* < 0.05). (e) Cell immunofluorescence staining *α*-actinin in the Ad-vector and Ad-miR-212 group. (f) The expression of miR-212 in the vector and miR-212 inhibitor groups (“∗” indicates statistical difference from the vector group *P* < 0.05). (g) The expression of ANP, myh6, and myh7 in the vector and miR-212-inhibitor groups (“∗” indicates statistical difference from the vector group *P* < 0.05).

miRNA with a high specific richness expressed in the heart often plays a crucial biological function in the occurrence and development of HF. The expression level of miR-212 was detected in the hypertrophy model, and it was found that the expression of miR-212 was increased in the rat model with abdominal aortic constriction for 4 weeks, and the expression of miR-212 was also dramatically increased in the model of mast cardiomyocytes induced by PE and Ang II (Figure 3(a)), suggesting that miR-212 may be involved in the regulation of CH. Then, adenovirus blank Vector (Ad-vector) was used as the control group, and miR-212 overexpressed adenovirus was transfected into cardiac muscle cells as the experimental group. Under the fluorescence microscope, we observed that most cardiomyocytes could emit strong green fluorescence, which proved that adenovirus transfection was effective (Figure 3(b)). We detected the expression level of miR-212 in the transfected primary cardiac myocytes by qRT-PCR, and the results showed that the expression level of miR-212 increased after the transfection of ad-miR-212 (Figure 3(c)), which proved that the construction of overexpressed adenovirus was successful. First, qRT-PCR was used to detect the changes in the expression of ANP, myh6, and myh7 in myocytes of the two groups. The results showed that after the same hunger stress, the myocytes transfected with Ad-miR-212 were dramatically higher in ANP and myh7 and decreased in myh6 compared with the control group (Figure 3(d)). Therefore, we hypothesized that miR-212 overexpression promoted primary CH. In addition, in the detection of cellular immunofluorescence, overexpression of miR-212 in primary cardiomyocytes promoted cardiomyocyte hypertrophy (Figure 3(e)). Compared with the PE-induced CH group, miR-212 expression was dramatically downregulated after PE-induced transfection with miR-212 inhibitor (Figure 3(f)). Moreover, ANP level was also reduced (Figure 3(g)), suggesting that knockdown of miR-212a expression in hypertrophic cardiomyocytes could effectively reverse the hypertrophy level of cardiomyocytes.

### 3.4. miR-212 Regulates CH by Targeting TCF7L2

**Figure 4:**
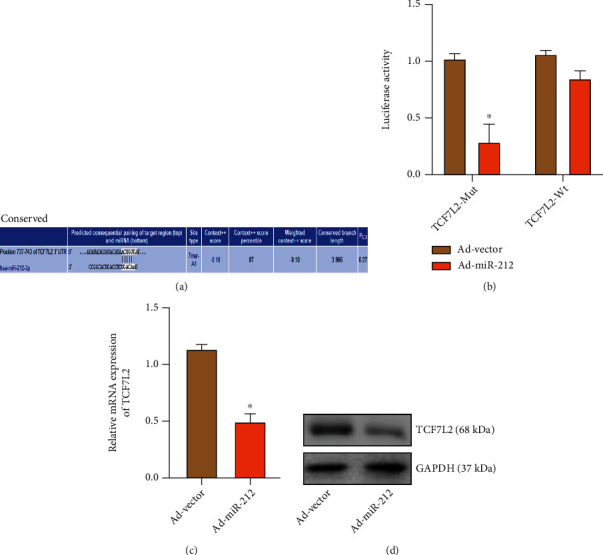
miR-212 regulates CH by targeting TCF7L2. (a) Results of site prediction. (b) Double-luciferase reporter gene assay (“∗” indicates statistical difference from the Ad-vector+TCF7L2-WT group *P* < 0.05). (c) The expression of miR-212 in the Ad-vector and Ad-miR-212 groups (“∗” indicates statistical difference from the Ad-vector group *P* < 0.05). (d) The band of TCF7L2 in the Ad-vector and Ad-miR-212 groups.

**Figure 5:**
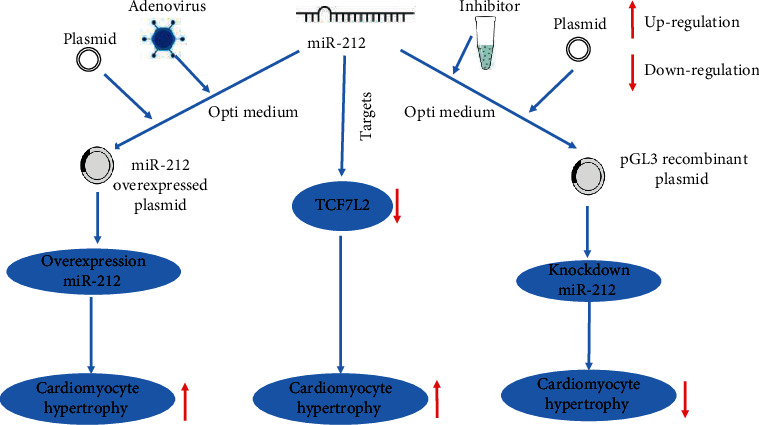
Schematic diagram of the molecular mechanism of miR-212 regulating CH by targeting TCF7L2.

We used the Targetscan website (https://www.targetscan.org/vert_80/) to predict the potential target genes of miR-212. Through retrieval, we found that rat TCF7L2 had the possible action sites of miR-148a in the 3′UTR region, and the matching situation is shown in the figure (Figure 4(a)). As a member of the T cell factor/lymphocyte enhancer factor (TCF/LEF) family, TCF7L2 is one of the downstream nuclear transcription factors of the classical Wnt/*β*-catenin signaling pathway and is involved in the regulation of cell proliferation and differentiation [18]. Recent studies have found a significant association between TCF7L2 gene polymorphism and diabetes mellitus. TCF7L2 is involved in the regulation of secretion, proliferation, and apoptosis of islet cells [19]. Then, the sequence of miR-212 interacting with TCF7L2 was inserted into the 3′UTR region of the reporter plasmid. After transfection with Ad-miRNA-212, 293T cells dramatically inhibited the luciferase expression at the TCF7L2 action site (Figure 4(b)). Next, we overexpressed miR-212 in primary cardiac myocytes and detected the expression changes of TCF7L2 by qRT-PCR and western blot. It was found that after overexpression of miRNA-212, the expression of TCF7L2 decreased (Figures 4(c) and 4(d)). Further evidence suggests that miR-212 may regulate CH by targeting TCF7L2 (Figure 5).

## 4. Discussion

We constructed a model of MH from the tissue to the cell level. The results verified each other and confirmed the success of the model construction; it can provide a good experimental basis and platform for further research on the expression and function of miRNA-212 in MH. At the same time, we further determined that the expression level of miR-212 was upregulated in the HF model. Then, the expression of miR-212 in cardiac myocytes was artificially interfered with by transfection with overexpressed adenovirus and inhibitor. However, knockdown of miR-212 can reverse the cell hypertrophy induced by PE to a certain extent. Then, we predicted the downstream target genes of miRNA-212 through the website and verified the regulation of miR-212 by targeting TCF7L2 on CH by luciferase gene reporting experiment, qRT-PCR, and WB.

Ventricular remodeling is the pathological basis of chronic HF. According to the results of this experiment, the rat heart showed obvious left ventricular hypertrophy and ventricular enlargement after 4 weeks of modeling. HE staining showed the characteristics of hypertrophy and disordered arrangement of cardiomyocytes, myocardial interstitial fibrosis, nuclear thickening, and increased myocardial sarcomere. In addition, the symptoms of HF in rats all indicated that the abdominal aortic model was a mature and reliable method. There are many causes of HF, and the choice of model is often decided according to the cause of the disease. Abdominal aortic constriction is due to CH caused by excessive pressure load, and the heart gradually goes from the compensatory stage to the decompensated stage. Therefore, the clinical diseases corresponding to this model are more similar to HF caused by hypertensive cardiomyopathy [20].

Myosin is an important part of the physiological structure of myocardium and also plays a crucial biological function; myosin heavy chain (MYHC) plays a vital role in cardiac contractile function. The encoded proteins corresponding to myh6 and myh7 are, respectively, a-MYHC and p-MYHC. Although these two genes are located on the same chromosome, their expression regulation is independent of each other. When cardiomyocytes age, due to changes in fetal gene expression, diastolic and contractile functions are reduced compared to normal cardiomyocytes. At the gene level, the expression of SERCA2 is reduced, myh6 is converted to myh7, and the expression level of ACTA1 is increased [21]. Since HF is often accompanied by changes in myocardial structure, the expression levels of myh6 and myh7 changes can also be a good representative of the changes in heart function.

Studies have shown that in the process of HF, a large number of miRNA expression is dysregulated, which plays a crucial role in regulating the process of myocardial remodeling. Correcting the expression of miRNA will have a significant reversal effect on MH. The use of miRNA as a diagnostic marker for HF in clinic has been widely reported [22]. Sinfield et al. proposed that miR-423-5p can be used as a diagnostic marker for HF [23]. In addition, miR-1, miR-133, miR-499, and miR-208 in acute myocardial infarction were all proved to be dramatically elevated, and further meta-analysis was performed to verify the role of miRNA [24]. The impaired cardiac contractility caused by the imbalance of calcium pump is an important sign in the process of HF. Studies have found that miR-765 is overexpressed in HF samples and can increase the activity of protein phosphatase PP-1 and increase the dephosphorylation of calcium circulating protein by inhibiting endogenous inhibitor-1. miR-25 is also believed to inhibit the calcium pump SERCA2a in HF and lead to HF [25]. Inhibiting the expression of miR-25 can effectively restore cardiac contractile function and improve survival rate of HF patients. Also, in the process of MH, miRNA may be used as a new biomarker for predicting and diagnosing HF. In this sense, the functional study of miR-212 is also important in clinical work. However, there are still some limitations in the present study. For example, we did not test whether knockdown or overexpression of miR-212 had an effect on myocardial hypertrophy in animal models. What is more, after we predicted TCF7L2 as a possible target gene of miR-212, we did not further verify the accuracy of the target gene by interfering with the expression of TCF7L2. In the future, our research group will further carry out the above unfinished experiments under the premise of sufficient funds, so as to better illustrate the clinical value of this research.

## 5. Conclusion

miRNA-212 targets TCF7L2 and inhibits the expression of this gene, possibly through this pathway to promote cardiomyocyte hypertrophy. The results of the current study may provide new insights for the treatment of cardiomyocyte hypertrophy.

## Figures and Tables

**Table 1:** Real-time PCR primers.

Gene name	Forward (5′>3′)	Reverse (5′>3′)
miR-212	AGCGTAACAGTCTCCAGTC	TCCTCCTCTCCTTCCTTCTC
ANP	GCTTCCAGGCCATATTGGAG	GGGGGCATGACCTCATCTT
myh7	ACTGTCAACACTAAGAGGGTCA	TTGGATGATTTGATCTTCCAGGG
myh6	GCCCAGTACCTCCGAAAGTC	GCCTTAACATACTCCTCCTTGTC
TCF7L2	AACGAACACAGCGAATGTTTCC	GACCTTGCCATCCTAGCGG
U6	GCTTCGGCAGCACATATACTAAAAT	CGCTTCACGAATTTGCGTGTCAT
GAPDH	ACAACTTTGGTATCGTGGAAGG	GCCATCACGCCACAGTTTC

RT-PCR: quantitative reverse-transcription polymerase chain reaction.

## Data Availability

The datasets used and analyzed during the current study are available from the corresponding author on reasonable request.

## Abbreviations

HF: Heart failure
HE: Hematoxylin-eosin
qRT-PCR: Quantitative real-time polymerase chain reaction
CH: Cardiac hypertrophy
MH: Myocardial hypertrophy
ncRNA: Noncoding RNA
lncRNA: Long noncoding RNA
circRNA: CircularRNA
miRNA: microRNA
AKT: Phosphatidylinositol 3 kinase (PI3K)/protein kinase B
BNP: Brain natriuretic peptide
SD: Sprague-Dawley
PBS: Phosphate-buffered saline
DMEM: Dulbecco's Modified Eagle's Medium
PE: Phenylephrine
RIPA: Radioimmunoprecipitation assay
BCA: Bicinchoninic acid
SDS-PAGE: Sodium dodecyl sulphate-polyacrylamide gel electrophoresis
PVDF: Polyvinylidene difluoride
GAPDH: Glyceraldehyde-3-phosphate dehydrogenase
ECL: Enhanced chemiluminescence
PBST: Phosphate-buffered saline-tween
cDNA: Complementary DNA
BSA: Bovine serum albumin
ATCC: American Type Culture Collection
RAAS: Renin-angiotensin-aldosterone system
ANP: Atrial natriuretic peptide
myh6: Myosin heavy chain 6
myh7: Myosin heavy chain 7
TCF/LEF: T cell factor/lymphocyte enhancer factor
MYHC: Myosin heavy chain.
